# Relationship between the Chromosome Structural Dynamics and Gene Expression—A Chicken and Egg Dilemma?

**DOI:** 10.3390/microorganisms10050846

**Published:** 2022-04-20

**Authors:** Diana Le Berre, Sylvie Reverchon, Georgi Muskhelishvili, William Nasser

**Affiliations:** 1 Université de Lyon, Université Claude Bernard Lyon 1, INSA-Lyon, CNRS, UMR5240 MAP, CEDEX, F-69622 Villeurbanne, France; diana.le-berre@etu.univ-lyon1.fr (D.L.B.); sylvie.reverchon-pescheux@insa-lyon.fr (S.R.); 2 School of Natural Sciences, Agricultural University of Georgia, 0159 Tbilisi, Georgia; g.muskhelishvili@icloud.com

**Keywords:** bacteria, genetic regulation, transcription, DNA supercoiling, chromosome dynamics, chromosomal expression domains, nucleoid-associated proteins

## Abstract

Prokaryotic transcription was extensively studied over the last half-century. A great deal of data has been accumulated regarding the control of gene expression by transcription factors regulating their target genes by binding at specific DNA sites. However, there is a significant gap between the mechanistic description of transcriptional control obtained from in vitro biochemical studies and the complexity of transcriptional regulation in the context of the living cell. Indeed, recent studies provide ample evidence for additional levels of complexity pertaining to the regulation of transcription in vivo, such as, for example, the role of the subcellular localization and spatial organization of different molecular components involved in the transcriptional control and, especially, the role of chromosome configurational dynamics. The question as to how the chromosome is dynamically reorganized under the changing environmental conditions and how this reorganization is related to gene expression is still far from being clear. In this article, we focus on the relationships between the chromosome structural dynamics and modulation of gene expression during bacterial adaptation. We argue that spatial organization of the bacterial chromosome is of central importance in the adaptation of gene expression to changing environmental conditions and vice versa, that gene expression affects chromosome dynamics.

## 1. Introduction

**Figure 1:**
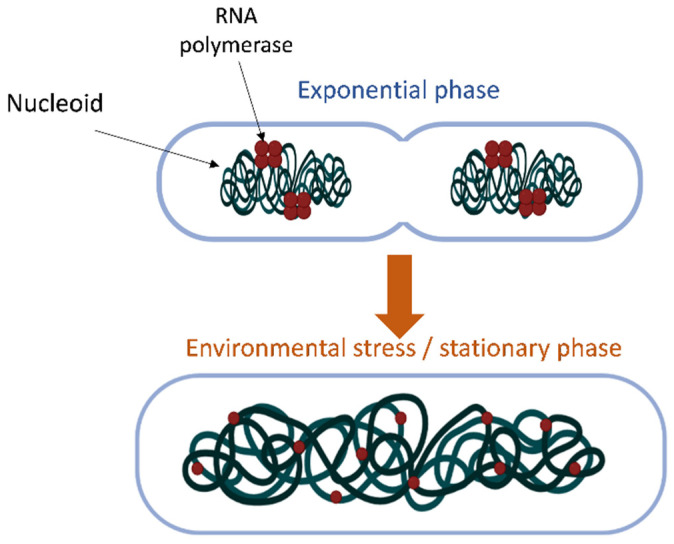
Localization of RNA polymerase (red) and cellular space occupied by the nucleoid (green) during rapid exponential growth and early stationary growth or stress conditions.

The organization of the bacterial nucleoid structure is a compromise between the requirements of the dramatic (about thousand-fold) compaction of DNA and the accessibility of the genetic material to enzymes securing vital functions such as replication, transcription and repair. Recent studies demonstrate that bacterial chromosomes are not only highly compacted but also spatially organized, with distinct genetic loci occupying specific positions in the cell [1,2,3,4]. In parallel with this organizational feature, the order of genes along the chromosomal replication origin to the terminus (OriC-Ter) axis was found to be highly conserved in bacteria [5]. This gene order appears to be highly relevant since recent observations suggest the limited diffusion of mRNAs from their site of production and the close physical coupling of translation to the site of transcription in the nucleoid [6,7]. In *Escherichia coli*, it is shown that chromosome compaction varies during the growth cycle. For example, during rapid exponential growth characterized by high levels of negative superhelicity, the chromosome is condensed with RNA polymerase (RNAP) mainly accumulated at a few highly active loci such as those encoding ribosomal RNA [8]; by contrast, at the entry in the stationary phase of growth associated with DNA relaxation, the chromosome decondenses demonstrating a uniform distribution of RNAP molecules over its entire surface (Figure 1). There is ample evidence that the different levels of chromosomal DNA organization play a role in transcriptional regulation and that conversely, transcription affects chromosome organization [9,10,11,12]. The relationship between chromosomal DNA supercoiling and transcription is aptly defined as a ‘two-way street” [13] which essentially poses a chicken and egg dilemma. Configurational changes of the chromosome (or particular chromosomal regions) appear to reveal an effective adaptation strategy for producing rapid coordinated genetic responses to adverse conditions [1,14]. Accordingly, the organization of nucleoid structure is hierarchical and dynamic due to the input of various factors, including transcription, molecular crowding, DNA supercoiling and the binding effects of nucleoid-associated proteins (NAPs) [4,9,15,16]. Of these factors, NAPs and transcription-generated supercoiling gained substantial attention during recent years.

## 2. NAPs Are Versatile and Flexible Actors in Coupling Nucleoid Organization and Transcription Request

Chief among the bacterial global regulators are the abundant NAPs, which are thought to influence both the chromatin structure and transcription [1,15,17,18]. Various studies using both ensemble and single-molecule experiments revealed that the binding of NAPs could result in the bending, bridging, wrapping and clustering of DNA [9,15]. Most NAPs bind DNA with relatively low specificity and form higher-order nucleoprotein complexes; notably, NAP binding sites are generally AT-rich, which is a characteristic feature of gene promoters. By virtue of their ability to constrain DNA supercoils, NAPs both facilitate the compaction of the bacterial chromosome and also exert long-range regulatory effects on genomic transcription [1,17,19], whereas transcription factors with more local effects essentially act upon the structural constraints imposed by NAPs. Both the abundance and the composition of NAPs as well as the global superhelical density of DNA, change with the growth phase [20,21]. Dynamic changes in chromosomal supercoil density during the bacterial growth cycle reflect the crosstalk between the NAPs and the DNA topoisomerases, which in turn depends on the metabolic state of the cell [22,23]. This crosstalk appears determinative for the growth phase-dependent pattern of gene expression [19]. Moreover, global supercoiling can also change instantly under the influence of environmental factors, optimizing the genomic transcriptional response to challenges [24,25]. This optimization involves the cooperative binding effects of various NAPs stabilizing distinct supercoil structures and interactions favoring the expression of relevant genes [1,14,26,27]. For example, both in the human pathogenic bacteria, *Salmonella enterica*, and in the plant pathogenic bacteria, *Dickeya dadantii*, cooperative effects of NAPs and DNA supercoiling coordinate the expression of virulence and adaptive genes required at different stages of pathogenic growth [28,29,30,31]. Notably, recent phylogenetic studies revealed that NAPs are highly conserved within bacterial families, some of them being conserved among all prokaryotic species [4,15] while all bacterial species encode at least one NAP [16], suggesting a selective advantage of NAP-dependent nucleoid structuring. 

**Figure 2:**
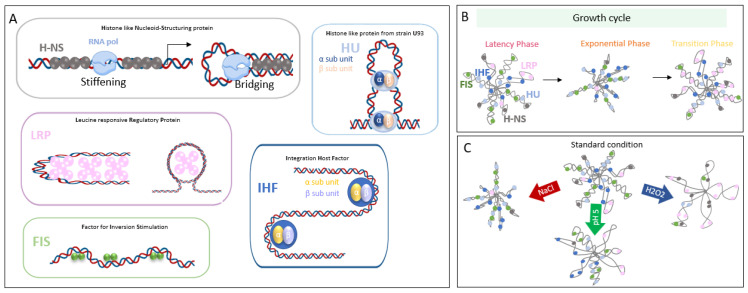
(**A**) Structural impact of the main Nucleoid-Associated Proteins (NAP) in DNA. (**B**,**C**) Changes in chromosomal compaction level and bound NAPs during growth (**B**) and under various environmental conditions (**C**).

NAPs thus play a crucial role in the adaptation of bacteria to unfavorable conditions and environmental stress [14,32,33,34]. Historically, in Gram-negative bacteria, the majority of studies on NAPs were performed in the model bacterium *Escherichia* *coli* and focused on the five major proteins H-NS, FIS, IHF, Lrp and HU, for which a direct role in transcriptional regulation was clearly established [15] and is briefly characterized below (Figure 2). 

### 2.1. H-NS

H-NS (histone-like nucleoid-structuring protein) is considered a global modulator of genes involved in adaptive processes related to changes in environmental conditions. DNA binding by H-NS is sensitive to environmental factors and DNA supercoiling states [1,15,30,35]. It binds cooperatively to AT-rich sequences to silence transcription [36,37]. Accordingly, H-NS controls virulence functions in a variety of animal and plant pathogens [30,38,39,40,41,42]. How H-NS represses gene transcription remains controversial. For some genes, H-NS is likely to occlude RNA polymerase binding, while for others, it represses transcription by binding downstream of the promoter [43]. Single-molecule experiments provide insights into how H-NS regulates gene expression by condensing the DNA. Atomic Force Microscopy (AFM) imaging reveals that H-NS can bridge adjacent DNA segments or form compact DNA-protein foci. The loops trapped by H-NS could, in some cases, prevent the binding of RNA polymerase or stall its translocation, while in some other cases, they trap the RNA polymerase open complex [43]. Further studies showed that H-NS could also extend DNA by polymerizing along the double helix in a Mg^2+^ concentration-dependent manner: above 5 mM Mg^2+^, the extension mode is inhibited, and instead, H-NS switches to bridging the DNA duplexes. It remains unclear whether H-NS primarily bridges or extends the DNA in vivo or whether these binding modes are mutually exclusive [43]. A more recent study revealed that H-NS-mediated environmental sensing operates mainly through its dimerization site2 located, as well as the dimerization site1, in the N-terminal domain of the protein [35,44]. The combination of site1 and site2 allows H-NS to multimerize and stably coat DNA with resultant gene silencing, whereas the unfolding of site2 at elevated temperature, high salinity or acidic pH results in an autoinhibited conformation incapable of stably interacting with DNA [35,45]. Interestingly, variations in the site2 sequence alter the sensing sensitivity of H-NS orthologs to fit different bacterial lifestyles [35].

### 2.2. FIS 

FIS (factor for inversion stimulation) is a NAP initially characterized as a stimulator of site-specific DNA recombination. FIS activates genes and operons involved in primary metabolism, including those encoding biosynthetic enzymes and stable RNAs [46]. FIS is also required for *oriC*-directed DNA replication and modulates the DNA supercoiling state by repressing DNA gyrase and stimulating topoisomerase I gene expression [47,48]. FIS can also influence DNA topology directly by binding to DNA and constraining supercoils. In particular, it acts as a buffer preserving intermediately supercoiled forms of DNA and precluding extreme shifts towards the more relaxed or more negatively supercoiled ends of the topological spectrum [49]. FIS is thus considered to act as a local topological homeostat [23]. The cellular concentration of FIS is subject to complex and multifactorial regulation. FIS is strongly produced during the exponential phase of cells grown in a rich medium and becomes nearly undetectable before the cells enter the stationary phase [50]. One important function of FIS is to shut off the expression of nonessential genes during rapid growth [51]. Furthermore, FIS is implicated in the regulation of virulence functions in pathogenic strains of *E. coli* [52], *Shigella flexneri* [53], *Salmonella* [54,55], *Vibrio cholera* [56] and *D. dadantii* [57]. 

### 2.3. IHF

IHF (integration host factor) is a heterodimeric protein composed of IHF α and IHF β subunits, respectively, encoded by *ihfA* and *ihfB* genes. Upon binding, IHF bend DNA by as much as 180 °C and thus promote long-range interactions. The influence of IHF on local DNA structure is critical for its contribution to transcriptional regulation [31]. In some cases, the DNA-bending activity of IHF is shown to enhance the formation of open complexes by facilitating the transmission of DNA twist from upstream AT-rich DNA regions to the promoter [58]. IHF also affects chromosomal replication initiation and DNA transposition. Therefore, IHF plays a role in both nucleoid structuring and DNA rearrangement. In addition, IHF is identified as a regulator of virulence functions in various pathogenic bacteria, including *Brucella abortus*, *Shigella flexneri*, *Salmonella enterica*, enteropathogenic and enterohemorrhagic *E. coli*, *V. cholerae* and *D. dadantii* [31,59,60].

### 2.4. HU

HU (histone-like protein from strain U93) is found as a homo- or heterodimer of the homologous subunits α and β. HU has a preference for binding distorted regions of the DNA, such as kinks or four-way junctions [15]. In vitro HU facilitates toroidal coiling of the DNA, which is apparently antagonized by H-NS [26]. HU can contribute to nucleoid compaction by bending or wrapping DNA [61]. Transcriptome studies revealed a role in the supercoil-constraining capacity of HU in gene regulation [62]. Furthermore, HU is proposed to modulate the gradient of DNA superhelical density and strength of transcription along the OriC-Ter axis of the chromosome [63]. HU modulates the expression of a large number of genes in *E. coli*, including those involved in primary metabolism and respiration [15]. HU also coordinates the virulence function and the stress response in *S. enterica* [34]. Recent findings implicate HU in remodelling the nucleoid architecture during bacterial growth and synchronizing the genetic responses mediated by variable constraints of DNA superhelicity under changing environmental conditions [61]. 

### 2.5. LRP 

Lrp (leucine-responsive regulatory protein) regulates the expression of more than 10% of the *E. coli* genome [15,64,65]; its activity can be modulated or left unaffected by leucine depending on the function of the target genes. The Lrp regulon includes genes involved in nutrient uptake and amino acid metabolism. Lrp is thought to mediate “feast and famine” transitions because of its opposite regulation of amino acid metabolism: activation of anabolic genes and repression of catabolic genes. Lrp also modulates bacterial virulence, regulating genes involved in the phase-variable expression of pili and nonfimbrial adhesin in *E. coli* and *Salmonella* [66,67] as well as LEE genes of *Citrobacter rodentium* [68]. It has a profound impact on the trajectory of the bound DNA. By wrapping DNA, Lrp can stabilize positive supercoils [15].

Thus, essentially, NAPs appear to coordinate metabolic demand and genomic expression (including the virulence ‘program’) in adaptation to the changing growth environment. However, NAPs are also involved in maintaining supercoiling homeostasis by modulating the activity of topoisomerases both via direct effects on their substrate topology (i.e., variable constraint of DNA supercoils) and indirectly by regulating topoisomerase gene expression.

## 3. Transcription, a Global Regulator of DNA Supercoiling State and Local Organization of the Nucleoid 

**Figure 3:**
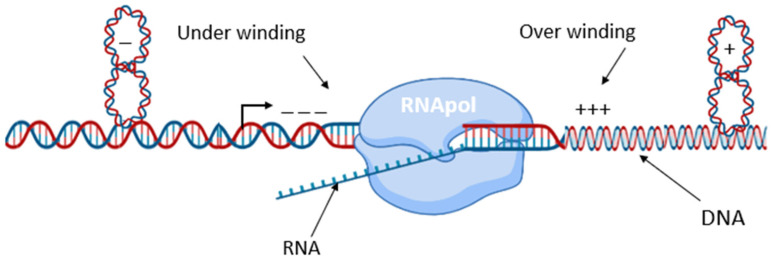
The twin supercoiling domain model. Transcription induces positive DNA supercoils downstream of RNA polymerase and negative DNA supercoils upstream. Diffusion of negative supercoiling behind RNA polymerase aids DNA melting and thus increases transcription initiation and inhibits termination, while diffusion of positive supercoiling in front of RNA polymerase inhibits initiation and increases termination.

Transcription is a complex multifactorial process in which the central player is RNA polymerase. The interconnections between the chromosome structural organization and RNA polymerase activity have been extensively reviewed [9,12,69,70,71]. It is long known that RNAP activity is modulated by sigma factors, NAPs, transcription factors and the structure of gene promoters [69]. Different RNAP holoenzymes demonstrate different preferences for supercoiling the template DNA [72]. However, the process of transcription itself significantly affects DNA topology by inducing positive supercoiling immediately downstream and negative supercoiling upstream of the translocating RNAP [73] (Figure 3). 

These transient changes in supercoiling can lead to supercoil diffusion modulating the activity of neighboring gene promoters [74]. Recent advances in high-throughput approaches and novel experimental technologies combined with bioinformatics tools provided a breakthrough in the field of transcriptional regulation. First, the studies revealed widespread antisense transcription in the genome [75]. Such antisense transcription resulted partially from the capacity of RNAP to remain bound to DNA following intrinsic termination and to restart transcription in the reverse direction [76]. Second, the relative spatial organization of the transcription units was implicated in gene regulation by relaying the DNA supercoil dynamics induced by translocating transcription machinery to neighbor genes over distances of ≥10 kb, substantially exceeding the size of individual operons [10,77,78]. Third, genomic spatial transcript patterns were observed that could not be explained on the basis of transcription factor–target gene interactions [78,79,80,81]. These findings underscored the importance of genetic control based on spatial considerations and, thus, ultimately, on the configuration of the chromosome.

On the other hand, compelling data was obtained concerning the dynamic organization of the bacterial chromosome. The positioning of the chromosome in growing cells was shown to follow a predetermined choreography. Furthermore, various dynamic organizational features of the *E. coli* chromosome structure pertinent to transcription were observed, including the *rrn* functional domain spanning the chromosomal Ori end, large clusters of genes sensitive to “nucleoid-perturbation”, DNA supercoiling-dependent spatial transcript patterns spanning regions from 16 to 800 kb in size, 10–20 kb topological domains and 5–10 kb gene proximity clusters [63,79,80,82,83]. Nevertheless, to date, chromosomal dynamics are mainly analyzed with respect to the processes of DNA replication and chromosome segregation [84,85,86], whereas the crosstalk between chromosome configuration and gene expression remains largely unknown. Here, we expand on this topic, with a focus on the coordination of bacterial gene regulation and chromosome dynamics.

## 4. Chromosomal Organization of an “Archipelago” of Functionally Related Genes Facilitates Coordinated Gene Transcription 

**Figure 4:**
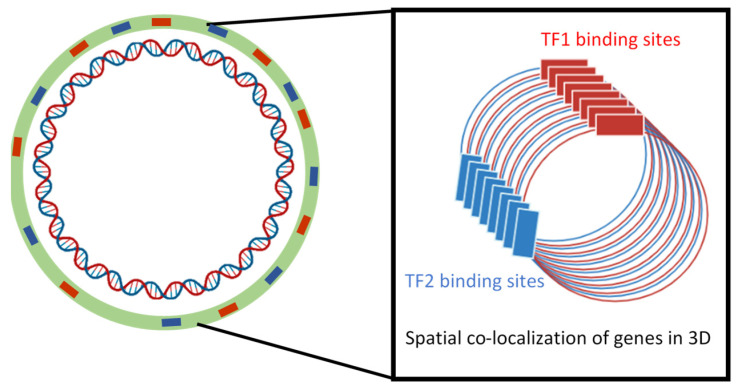
Spatial organization of genes scattered along the genome can result in archipelagos of co-expressed genes.

In order to reduce the energy spent on gene regulation and orchestrate the production of numerous factors involved in a metabolic pathway or adaptation to a particular challenge, bacteria have set up different synchronization tools and processes. For a long time, studies focused on genetic processes that elucidated the synchronization of genetic expression based on operon structures or the organization of genes in spatial proximity to each other (synteny). In contrast, the coordinated expression of regulons and, in general, coordinated expression of gene clustered at different places in the genome (‘archipelago’ organization) has long remained a mystery, confounded further by the emergence in the 2000s of data indicating a relatively slow diffusion of regulatory proteins from their sites of production in the cell. Consequently, epigenetic-type mechanisms linked in particular to the structural dynamics and organization of the chromosome previously observed in eukaryotes were then explored. Hence, the implementation of tools to assess the long-range patterns on a genome-wide scale prompted the study of co-regulated, isofunctional and evolutionary correlated gene sets [87,88,89,90]. The implementation of these tools enabled the proposal of models for the co-regulation of genes, especially those involved in secondary metabolism or virulence, scattered in the bacterial genome (Figure 4). 

However, the most successful model concerns the genes for pectinolysis in bacterial genera *Dickeya* and *Pectobacterium,* responsible for soft rot disease in a wide range of plants. In these bacteria, the major virulence factors are pectate lyases, secreted by a specific type II secretion system [91,92]. The genes encoding pectate lyases, the secretion system, and all the other cellular proteins involved in pectin catabolism are co-regulated by the KdgR transcription factor, which is responsible for their induction in the presence of pectin [93,94]. All these co-regulated genes are scattered in multiple islands in the genome, raising the question of the control of the timing of their synthesis and translocation. By using the so-called algorithm “patterns”, allowing the identification of the periodicity of gene location on the chromosome [87], found that KdgR targets are distributed periodically, being organized in a single “archipelago” on a genome-wide scale. Furthermore, the analyses revealed that the genes encoding secreted pectinases are expressed from the same DNA strand. Such organization is supposed to favor the funneling of newly synthesized pectinases toward a convergent point in the cell, optimizing their coordinated secretion via the type II secretion system. However, these bioinformatics analyses need to be validated experimentally, which can be conducted, e.g., by positionally shifting the *kdgR* gene in the chromosome, or by disrupting the periodic spatial distribution of the KdgR regulon by introducing DNA ‘spacers’ between the regulated genes (Figure 4). 

Chromosomal position shifts of regulatory genes are instrumental in gaining insights into spatial aspects of gene regulation. For example, the data obtained in *E. coli* revealed that the regulation of gene expression by the major NAP H-NS depends on the chromosomal position of the target promoters, indicating that genomic position can play a significant role in the adaptation of the cells to environmental changes [4,95]. Similar experiments using a positional shift of the *fis* gene encoding the NAP FIS demonstrated that spatial separation between regulator and target genes could influence their interaction and, ultimately, the bacterial phenotype [96]. Such spatial effects are consistent with the proposal that the physical structure of the chromosome is optimized by direct regulatory interactions involving the NAPs and other DNA structuring proteins [97,98]. Indeed, a comparison of the NAP effects on the frequency of DNA–DNA (Hi-C) interactions in *E. coli* [99] revealed that the various NAPs contribute to nucleoid organization by promoting or restricting long- and short-range contacts on a genome-wide scale. Recently [100], combined the study of gene expression and spatial organization (RNA-seq and Hi-C) of the genome with the computational analysis of the impact of chromosome spatial organization on gene expression in *E. coli*. They observed recurrent sinusoidal patterns with region-dependent frequencies and high co-expression proportional with spatial proximity (Figure 4). Computational data analysis and simulation, including the search of connection to specific binding profiles of various regulators, led the authors to postulate local effects of nucleoid structure in synchronizing gene expression. These synchronizing effects occur through transcriptional spilling onto neighboring genes by sharing the local pools of RNA polymerase. The proposed mechanism is thus intrinsically dependent on the cooperation between the three-dimensional organization of the nucleoid and local availability of RNA polymerase. However, the approach used by the authors did not allow the evaluation of the possible indirect effects of the tested regulators. This model would benefit from performing expression experiments simultaneously in the parental *E. coli* strain and in the strains inactivated for the relevant regulators. Similarly, experiments aimed at testing the effects of disrupting the spatial proximity of co-expressed genes would be useful to support the proposed model.

## 5. Impact of Genomic Sequence Organization and Physical Locations of Genes on Transcription

**Figure 5:**
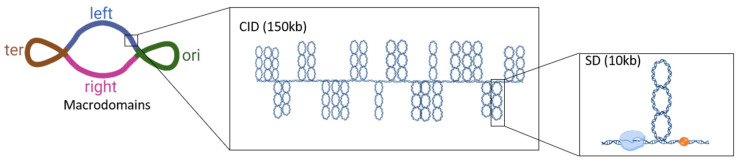
*Escherichia coli* chromosome organization. The chromosome is organized into 4 macrodomains with greater internal interactions: origin of replication, terminus of replication, the right and the left macrodomains. These macrodomains include more highly interacting chromosomal interaction domains (CIDs) and topologically isolated supercoiled domains (SDs).

Recent high-throughput studies mostly performed in *E. coli* and related bacteria discovered coherent transcript patterns spanning chromosomal regions from several kilobases to several hundred kilobases in size. Various factors are implicated in delimiting these regions of coherent gene expression, including strong transcription units [101,102,103], regulatory interactions [97], binding of NAPs and other DNA structuring proteins, including SMC proteins and macrodomain-specific proteins [99,104,105], DNA topoisomerases [4,71] and peculiar sequence organization [5,106,107]. We note that at the shortest length scales, the nucleoid is shown to be organized into supercoiled domains (SD) of ~10 kb in size that are topologically insulated from each other, and their existence is further supported by electron microscopy [83]. At a larger scale, Chromosome Conformation Capture (3C) and associated technologies (such as 3C-genomic and Hi-C approaches) revealed the existence of Chromosomal Interaction Domains (CIDs) as regions displaying high interaction frequencies [108]. A proportional relationship between the transcription level of genes and their contact frequencies was recently proposed in *E. coli* [99], implying that highly transcribed genes are organized spatially such that they interact more often. CIDs were reported for *Caulobacter crescentus*, *Bacillus subtilis* and *Mycobacterium pneumoniae*. They range in size from 30 to 400 kb and exhibit significant variability in boundary sharpness, indicating that different cells might have different, perhaps transient, domain borders. The genomic DNA within each CID seems to be organized into supercoiled plectonemic loops of at least 10-kb in size. Boundaries between the CIDs are frequently created by long and strongly transcribed genes, although not all highly transcribed genes are associated with boundaries [109]. In *C. crescentus*, it was shown that active transcription blocks supercoil diffusion but that not all supercoil diffusion barriers are CID boundaries [110]. Thus the molecular mechanisms for boundary formation remain unclear. The current model proposes that transcription drives the local decompaction of chromosomal DNA, lowering the contacts between neighboring regions and thereby forming domain boundaries [111]. This assumption is supported by the fact that the inhibition of transcription by rifampicin causes a dramatic loss of the CID boundaries [99,112,113]. Additionally, in *Mycoplasma*, a bacterium with a reduced genome, the chromosome appears to be organized in domains of 15–33 kb in size and the genes within the same domain tend to be coregulated, suggesting that chromosome organization influences transcriptional regulation [113]. However, no direct evidence supporting this hypothesis is provided and the causal relationships between the observed correlations are not elucidated (Figure 5). 

Recent studies of the higher-order architecture of the *E. coli* and *B. subtilis* genomes highlighted the role of NAPs in determining the chromosome configuration [99,114]. In *E. coli*, HU and FIS promote long-range chromosomal contacts, whereas H-NS restricts short-range interactions. However, the underlying mechanisms utilized by these NAPs remain unknown. The abundant NAPs can either constrain supercoiling within CIDs or SDs to relieve torsional stress and stabilize domains or create complexes bridging the domains, thereby forming a larger CID with nested SDs [9]. Interestingly, in *B. subtilis* it was recently reported that the H-NS-like DNA binding protein Rok forms large nucleoprotein complexes that robustly interact with each other over large distances forming anchored chromosomal loops. These spatially isolated large sections of DNA resemble those generated by insulator proteins in eukaryotes. Although the formation of these DNA loops has an impact on the dynamics of the CIDs, their significance remains to be elucidated [114]. Furthermore, as observed for H-NS in *E. coli*, Rok restricts short-range interactions consistent with its silencing function of gene expression, as short-range chromosome interactions often correlate with gene expression in bacteria [99]. 

On a larger scale, chromosomes are organized into several macrodomains (around 1 MB each), first documented in *E. coli* [115,116]. These domains are: the origin (Ori), Right, terminus (Ter) and Left macrodomains, as well as two additional non-structured domains, named the non-structured right (NSR) and left (NSL) domains that are, respectively, located between the Ori and the Right and Left macrodomains. Macrodomains were also identified in *B. subtilis* [112] but not in *Caulobacter* [117]. The macrodomains seem to be differentially regulated [5] and play functional roles in various DNA-related transactions such as replication and transcription [109]. Their properties seem to depend in part on active transcription, especially during the exponential growth phase [118]. Overall, it appears that both transcription and chromatin architecture influence domain formation, but the connection between the macrodomains, the CIDs with nested SDs and the process of transcription remains to be elucidated. It is necessary to explore additional bacterial models in various experimental conditions by evaluating, for example, the impact of factors implicated in the chromosome structuring under conditions of variable (high and low) transcription activity. 

Recent studies highlighted the importance of the transcription-coupled diffusion of supercoils (TCDS) and the formation of the so-called coherent domains of transcription (CODOs) spanning chromosomal regions from several tens to hundreds of kilobases and encoding particular traits [71,107]. The features of the CODOs resemble those of the well-known pathogenicity islands in which the grouped genes seem to be activated or repressed depending on the spatial pattern of organization, supercoil diffusion and regional structural dynamics, which directly correlate with DNA sequence organization. The CODOs were identified based on the physical properties of the expressed gene sequences, such as their dynamical behavior (supercoiling response), thermodynamic stability (average negative melting energy) and spatial orientation (leading/lagging strand bias). These domains of coherent transcription were particularly studied in the model commensal bacterium *E. coli* and the plant pathogenic bacterium *D. dadantii*. It was found that in *E. coli*, the genomic sequence organization favors a temporally sequential expression of genes required for growth along the OriC-Ter axis. Thus, the CODOs comprising relatively G/C rich genes required for rapid growth under conditions of high levels of negative DNA supercoiling are located in the vicinity of the origin, whereas those CODOs comprising relatively A/T rich genes less dependent on negative DNA supercoiling and required under conditions less favorable for growth are located closer to the terminus. In *Dickeya,* a more nuanced sequence organization of the genome is observed, demonstrating CODOs comprising genes coherently responding to different kinds of stress encountered during the infection process. It turned out that in *D. dadantii* various constellations of CODOs form transiently in response to particular environmental stress, whereby the CODOs demonstrate unique couplings between the dynamical and physicochemical properties of the expressed sequences, their functional content and the specific impacts of H-NS, FIS and IHF. Interestingly, while the mechanistic role of H-NS and FIS in the establishment of CODOs in *D. dadantii* remains unspecified, the establishment of CODOs by IHF was associated with the DNA supercoiling-dependent modulation of the directionality of transcription selecting the particular orientation of the transcribed genes [31]. Notably, inactivation of any of these three NAPs resulted in a reorganization of the genomic expression, thus attenuating bacterial pathogenicity by affecting many virulence genes required during the infection process [31].

Importantly, the targeted insertions of the *E. coli fis*P-YFP reporter construct in different CODOs in *D. dadantii* demonstrated that under various growth conditions, the reporter construct behavior reflected that of the targeted CODOs [107]. These observations suggested that CODOs are able to synchronize the expression of the inserted xenogenic promoter with the expression of functionally linked genes (including the virulence genes) comprised in the CODO [71,107]. The hypothesis that the DNA context has an impact on reporter gene transcription was subsequently supported by a study using high-density insertions of reporter genes at different locations in the *E. coli* chromosome. The quantification of reporter activities revealed a 20-fold variation in transcriptional propensity across the genome leading the authors to propose that this bacterium possesses reporter gene-independent mechanisms for regulating expression from specific chromosomal regions [119].

## 6. Possible Impact of Transcription on Subcellular Localization of Genes

**Figure 6:**
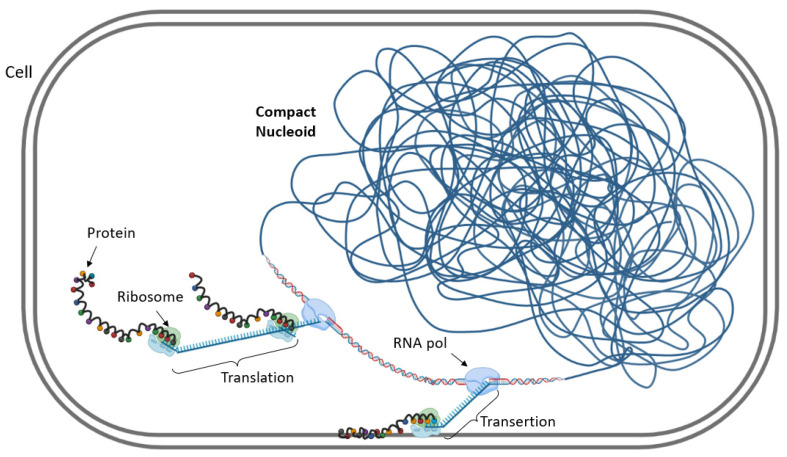
Impact of transcription on subcellular localization of genes. Transcription starts inside the nucleoid with formation of the RNAP–promoter complex. When the ribosome binding site of mRNA is transcribed, free ribosomal subunits in the nucleoid region bind the mRNA. The DNA–RNAP–mRNA–ribosome complex moves at the periphery of the nucleoid for easy ribosome access. Some membrane proteins are produced by coupled transcription–translation membrane insertion, a process refered to as transertion and responsible for chromosome anchoring to the inner membrane.

As mentioned above, the relationship between chromosomal DNA supercoiling and transcription is defined as a “two-way street” [13], meaning that these two processes are interdependent. Transcription has a direct impact on DNA topology as the transcribing RNAP induces positive supercoiling immediately downstream and negative supercoiling upstream of the transcription complex [73,120]. Furthermore, genes are often more efficiently transcribed from more underwound negatively supercoiled than from the more overwound positively supercoiled DNA due to the facilitated untwisting of the double helix and increased exposure of the promoter region on negatively supercoiled DNA [9,121]. Thus, the supercoil diffusion induced by transcribing RNAP can not only distinctly modulate the expression of the neighboring genes [74,122] but also act as an evolutionary force determining chromosomal gene arrangement [10]. The connection between transcription and nucleoid structure was most clearly exposed by combining molecular genetic approaches with high-resolution fluorescence microscopy. In particular, in both *E. coli* and *Bacillus subtilis* cells grown in rich media, it was reported that the accumulation of RNA polymerase molecules forms dense aggregates, so-called transcription foci, mostly engaged in rRNA synthesis [123,124,125]. Further investigations revealed that these clusters are preferentially enriched at the surface of the chromosome, leading to the proposal that actively transcribed regions are located at the periphery of the nucleoid [126]. A potential explanation for this is that while after transcription initiation, the nascent mRNA nucleates the recruitment of ribosomes, the resultant large DNA−RNAP−ribosome complexes are entropically excluded from the bulk of the DNA and tend to migrate to the nucleoid periphery [127,128] (Figure 6). 

However, this hypothesis was questioned by recent studies, which revealed that RNAP is able to form clusters in the presence of a limited number of rrn operons or in the absence of high levels of rRNA synthesis [11] and that the inhibition of gyrase activity leading to global DNA relaxation resulted in the redistribution of the RNAP clusters. It was thus postulated that it is primarily the underlying nucleoid structure rather than active transcription that directs the spatial distribution of RNA polymerase at the global level [11]. Notably, the organization of transcription foci requires the NAP HU, which modulates the genome-wide transcription [63] and is involved in both the maintenance of chromosomal supercoiling and facilitating the action of DNA gyrase [129,130]. Thus, HU is coordinately affecting the DNA supercoiling and chromosomal structure as well as gene expression. Given the direct impact of transcription on supercoiling [13,73,120] on the one hand, while the transcriptional activity of the RNAP holoenzyme depends on DNA superhelical density [23,72,131,132] on the other, it appears that NAPs, by virtue of their ability to constrain various supercoil structures, play a crucial role in coordinating chromosome configuration dynamics and genomic expression [17,19,133,134].

## 7. Conclusions 

There is growing evidence that the different levels of DNA organization play roles in transcriptional regulation and that, in turn, transcription affects chromosome organization [1,2,3,4]. Thus, transcription and bacterial chromosome dynamics are interconnected and influence each other according to physiological and environmental conditions. Nevertheless, the role of nucleoid structural dynamics in changing the gene expression underlying the capacity of bacterial species to quickly adapt to altered environmental conditions is often underestimated. Further investigations are required to fully decipher the mechanistic basis of interconnections between transcription and chromosome structural dynamics, in particular, to clearly demonstrate their tight interdependence and reciprocal impact and identify the conditions and circumstances under which the action of one predominates over the other. The advent of powerful new instruments, methodologies and informatics tools for the deep analysis of complex data now give an extraordinary opportunity to advance in this important field dealing with the exploration of fundamental devices coordinating bacterial genetic expression.
